# Monkeypox: Is it the new pandemic?

**DOI:** 10.1002/hsr2.963

**Published:** 2022-11-29

**Authors:** Imaduddin Sawal, Rabbia Tariq, Saleha Bint‐e‐Shuaib, Asad Ali Khan, Irfan Ullah, Abdulqadir J. Nashwan

**Affiliations:** ^1^ Dow University of Health Sciences Karachi Pakistan; ^2^ Internal Medicine, Hayatabad Medical Complex Peshawar Pakistan; ^3^ Kabir Medical College Gandhara University Peshawar Pakistan; ^4^ Institute of Public Health and Social Science (IPH&SS) Khyber Medical University Peshawar Pakistan; ^5^ Hamad Medical Corporation Doha Qatar

**Keywords:** COVID‐19, monkeypox, pandemics, zoonotic

## 1 INTRODUCTION

**Figure 1:**
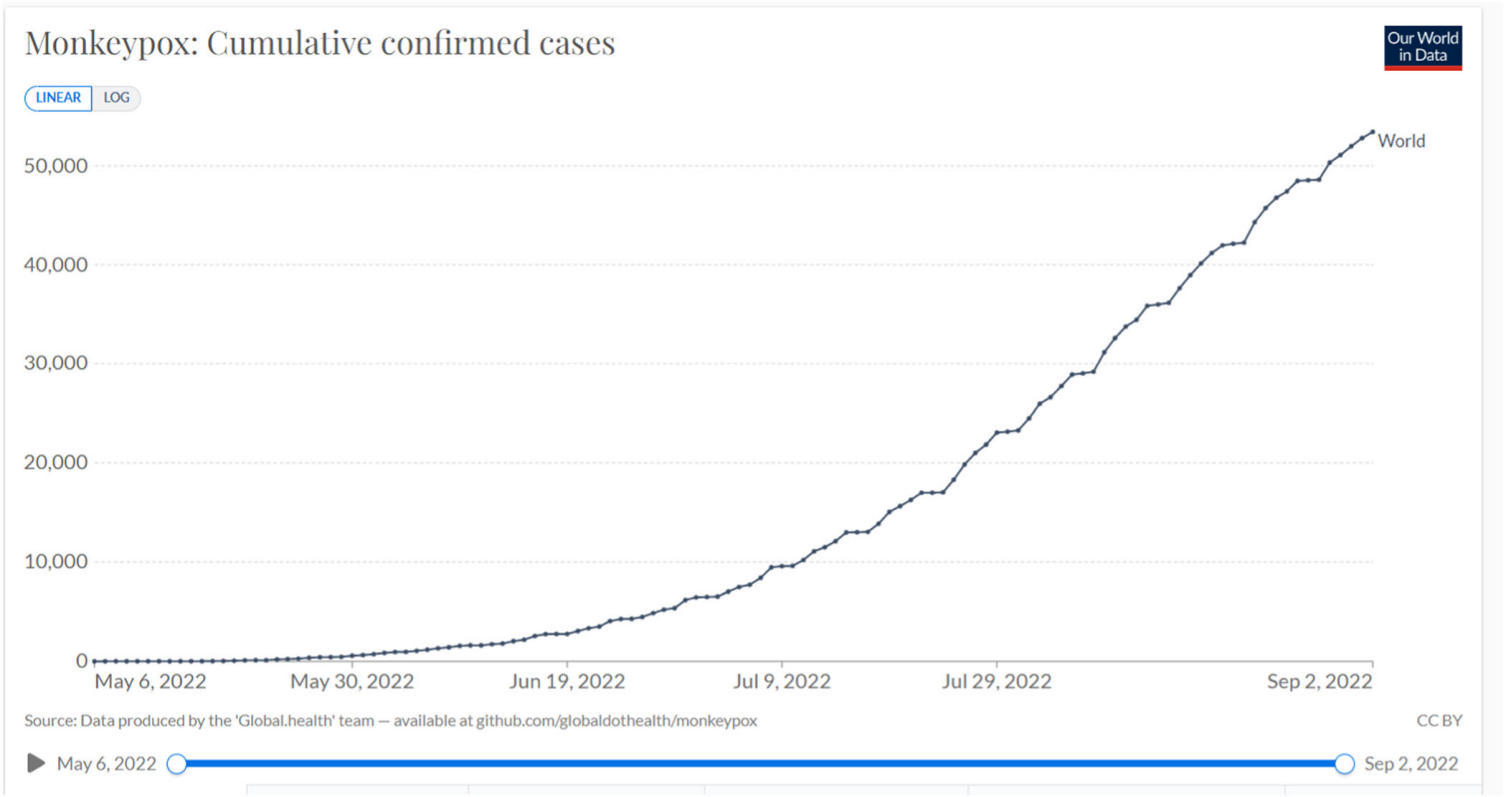
Monkeypox cumulative number of confirmed cases

Since the start of the monkeypox (MPX) outbreak and as of September 1, 2022, at least 18,463 confirmed cases of MPX have been reported worldwide (Figure 1).^1^

The WHO Director‐General declared MPX as a Public Health Emergency of International Concern (PHEIC) on July 23, 2022. Some scientists have commented that the surge in cases was an outbreak that was bound to happen after the end of vaccination against smallpox globally around 40 years ago.^2^ The head of the smallpox team, a part of the WHO Emergencies Programme, said that what is unusual about this current outbreak is that the countries usually spared from MPX are now reporting outbreaks of the virus.^3^

## 2 HOW DID IT START, CURRENT, AND EXPECTED PROGRESSION?

The current outbreak began with the first case being confirmed on 6th May 2022 when a British resident who had traveled from Nigeria on 4th May presented symptoms of the MPX virus. However, a while later, it was reported that out of the seven cases, only the first had a recent travel history to Africa.^4^ This indicates that community transmission is taking place. Some experts commented that four of the seven cases seen in England in the past 2 weeks were found in homosexual or bisexual men, and this is suggestive of sex being a driver of transmission.^4^ However, others are doubtful of this theory, especially because there's no history of MPX being sexually transmissible.^4^ The current outbreaks are worrying because there has not been a precedence of extended chains of human‐to‐human spread of the virus before. So, there are a lot of unanswered questions on whether the virus has evolved or whether it results from a loss in the population‐level immunity to poxviruses.

## 3 DISEASE, SYMPTOMS, AND MORTALITY

Human MPX is a zoonotic illness caused by the MPX virus, a dsDNA virus belonging to the Orthopoxvirus genus of the Poxviridae family. MPX is the most common orthopoxvirus infection in humans since the global elimination of smallpox in 1977.^5^ MPXV is a linear DNA genome of around 197 kb and contains nearly 190 nonoverlapping open reading frames ORFs >180 nucleotides long.^1^ One study predicts that four lineages are circulating in the reservoir population currently.^1^ It was initially isolated and identified following an occurrence in which monkeys being moved from Singapore to a Danish research laboratory became sick.^6^

That's how the virus got to be named “monkeypox.” The illness is especially common in Central and Western Africa; a variety of rodents and nonhuman primates serve as animal hosts.

The virus is spread by direct or indirect contact with skin lesions, body fluids, or respiratory droplets of infected animals.^5^ The symptoms initially include pyrexia, headache, fatigue, and lymphadenopathy, which is a symptom that mainly sets apart MPX from smallpox.^5^ After a few days, mucosal lesions appear in the mouth, followed by skin sores on the limbs and face. The total number of lesions may range from a few to several thousand.^3^ Other skin disorders, including as measles, chickenpox, scabies, and bacterial skin infections, should be investigated as differential diagnosis. According to the American Society of Microbiology, polymerase chain reaction is the preferred laboratory test for MPX diagnosis. Using rapid antigen and serology is of limited value because the virus demonstrates cross‐reactivity with other orthopoxviruses.

MPX symptoms usually disappear on their own without the need for treatment. According to the WHO, the death rate of MPX in the general population has traditionally fluctuated from 0% to 11%, with small children being more vulnerable. Recently, the case fatality ratio has varied between 3% and 6%.^3^

## 4 THE IMPACT OF EARLIER MPX OUTBREAKS

This is not the first such outbreak in recent history. A similar MPX outbreak previously happened in 2003 in the United States, the first outside Africa. In the spring of 2003, smallpox‐like rash cases were reported in the Midwest. This grew to 71 cases in six states by July. The case's origin was later found to be pouched rats imported from Ghana. No deaths resulted from the outbreak.^7^

## 5 THROWBACK TO THE CURRENT SCENE!

Although smallpox and MPX viruses are different, they are similar enough that the vaccine for smallpox protects against infection from both. The smallpox vaccine is 85% effective at preventing MPX based on observational studies in Africa, according to the WHO and the CDC. In 2019, a newer two‐dose vaccine containing a modified vaccinia virus was approved to prevent MPX. However, it is still not widely available.^8^, ^9^ The risk of developing MPX may be exceptionally high in people born after smallpox vaccination campaigns were discontinued. Therefore, this vaccine could be a game changer due to its effectiveness.

Apart from preemptively vaccinating those in places of the outbreak, people who were in contact with those spreading MPX could be vaccinated, in a process known as **“ring vaccination.”** Since the disease can be transmitted from direct contact and contaminated items, extra care needs to be exercised when dealing with suspected or confirmed MPX patients, ensuring proper personal protective equipment and regular changing of bedsheets and equipment. Correct information regarding the virus, such as symptoms and prognosis, must be spread through conventional and digital media. The stigma of the disease being associated with a particular group of people should be tried to be erased, and an early check‐up and diagnosis should be encouraged.^8^

## 6 HOW ARE COUNTRIES ALREADY PREPARING FOR IT?

Some countries have already started piling up vaccines for a potential virus outbreak. Germany, for example, announced that it has ordered up to 40,000 doses of a vaccine used to treat smallpox to be ready in case the outbreak takes a turn for the worse.^10^ An infectious disease specialist at the CDC said that a crucial difference between MPX and SARS‐COV‐2 is that MPX is a known entity and we are not at square one when facing MPX compared to coronavirus, and that's the positive news. However, the trajectory of the outbreak still remains uncertain.^11^

## 7 CHALLENGES

However, as seen previously in the COVID‐19 pandemic,^12^ a major foreseeable problem might be the unequal distribution of smallpox vaccines, where financially stronger countries hoard vaccines. In contrast, the countries most affected are left with a deficit of doses.

## 8 RECOMMENDATIONS

A few steps are essential in preventing the spread of MPX further. One way to reduce its spread is by identifying cases and possible outbreak areas by ramping up testing capacity. This can be done by promoting awareness and providing easier and affordable access to testing. Healthcare professionals need to be taken in the loop for complete and the most recent knowledge of the disease. This will enable them to better manage the patients and also reduce the risk of transmission to other patients and doctors by exercising proper infection prevention precautions if a diagnosis of MPX is being considered. Vaccination can also be quite helpful. Two types of orthopoxviruses are available, a live modified vaccinia Ankara (MVA0 vaccine), and a replication‐competent smallpox vaccine. According to one study, vaccinated people had a fivefold lower risk of MPX as compared with unvaccinated persons, and vaccine efficacy was estimated to be approximately 81 percent in those with a distant history of smallpox vaccination.^13^ Vaccination is recommended as pre‐exposure prophylaxis especially in people at risk for orthopoxvirus infection, for example, because of their occupational setting or sexual risk.^13^

## 9 CONCLUSION

All in all, the MPX virus does not seem to be as deadly as the earlier COVID‐19 pandemic. However, that is only possible if all affected countries exercise proper caution and strict measures are taken. If taken lightly, the disease may significantly affect more people and may wreak a similar effect as the COVID‐19 pandemic.

## AUTHOR CONTRIBUTIONS

**Imaduddin Sawal**: Writing – original draft; writing – review and editing. **Rabbia Tariq**: Writing – original draft; writing – review and editing. **Saleha Bint‐e‐Shuaib**: Writing – original draft; writing – review and editing. **Asad Ali Khan**: Writing – original draft; writing – review and editing. **Irfan Ullah**: Writing – original draft; Writing – review and editing. **Abdulqadir J. Nashwan**: Writing – original draft; writing – review and editing.

## CONFLICT OF INTEREST

Abdulqadir J. Nashwan is an Editorial Board member of Health Science Reports and coauthor of this article. He is excluded from editorial decision‐making related to the acceptance of this article for publication in the journal. The authors declare no conflict of interest.

## TRANSPARENCY STATEMENT

The lead author Abdulqadir J. Nashwan affirms that this manuscript is an honest, accurate, and transparent account of the study being reported; that no important aspects of the study have been omitted; and that any discrepancies from the study as planned (and, if relevant, registered) have been explained.

## Data Availability

All data generated during this study are included in this published article.
